# Rural populations facilitated early SARS-CoV-2 evolution and transmission in Missouri, USA

**DOI:** 10.1038/s44298-023-00005-1

**Published:** 2023-12-05

**Authors:** Cynthia Y. Tang, Tao Li, Tricia A. Haynes, Jane A. McElroy, Detlef Ritter, Richard D. Hammer, Christopher Sampson, Richard Webby, Jun Hang, Xiu-Feng Wan

**Affiliations:** 1 Center for Influenza and Emerging Infectious Diseases, University of Missouri, Columbia, MO USA; 2 Molecular Microbiology and Immunology, School of Medicine, University of Missouri, Columbia, MO USA; 3 Bond Life Sciences Center, University of Missouri, Columbia, MO USA; 4 Institute for Data Science and Informatics, University of Missouri, Columbia, MO USA; 5 Viral Diseases Branch, Walter Reed Army Institute of Research, Silver Spring, MD USA; 6 Family and Community Medicine, University of Missouriś, Columbia, MO USA; 7 Anatomic Pathology & Clinical Pathology, University of Missouri, Columbia, MO USA; 8 Emergency Medicine, University of Missouri, Columbia, MO USA; 9 Infectious Diseases, St. Jude Children’s Research Hospital, Memphis, TN USA; 10 Department of Electrical Engineering & Computer Science, College of Engineering, University of Missouri, Columbia, MO USA

**Keywords:** Epidemiology, SARS-CoV-2, Viral epidemiology, Viral evolution, Viral transmission

## Abstract

In the United States, rural populations comprise 60 million individuals and suffered from high COVID-19 disease burdens. Despite this, surveillance efforts are biased toward urban centers. Consequently, how rurally circulating SARS-CoV-2 viruses contribute toward emerging variants remains poorly understood. In this study, we aim to investigate the role of rural communities in the evolution and transmission of SARS-CoV-2 during the early pandemic. We collected 544 urban and 435 rural COVID-19-positive respiratory specimens from an overall vaccine-naïve population in Southwest Missouri between July and December 2020. Genomic analyses revealed 53 SARS-CoV-2 Pango lineages in our study samples, with 14 of these lineages identified only in rural samples. Phylodynamic analyses showed that frequent bi-directional diffusions occurred between rural and urban communities in Southwest Missouri, and that four out of seven Missouri rural-origin lineages spread globally. Further analyses revealed that the nucleocapsid protein (N):R203K/G204R paired substitutions, which were detected disproportionately across multiple Pango lineages, were more associated with urban than rural sequences. Positive selection was detected at N:204 among rural samples but was not evident in urban samples, suggesting that viruses may encounter distinct selection pressures in rural versus urban communities. This study demonstrates that rural communities may be a crucial source of SARS-CoV-2 evolution and transmission, highlighting the need to expand surveillance and resources to rural populations for COVID-19 mitigation.

## Introduction

The rapid evolution of the severe acute respiratory coronavirus 2 (SARS-CoV-2), the causative virus of the coronavirus disease 2019 (COVID-19), has resulted in the emergence of multiple variants with increased virus transmissibility^1–3^, immune evasion^4–8^, and pathogenicity^9,10^. Ease of travel has allowed new variants to quickly spread from essentially any location to a global scale^11^. Consequently, while emerging SARS-CoV-2 variants are often detected in high-resource settings with genome sequencing capabilities, their origins remain poorly defined. Further complicating the control of the virus is the lack of genomic surveillance among rural populations. Rural populations, comprising 20% of the US population^12^, have been particularly vulnerable to COVID-19 complications, including higher incidences of disease and mortality^13–20^, largely due to limited access to healthcare and hospital capacities compared to their urban counterparts^21,22^. By December 8, 2020, cumulative rural cases in America surpassed 2.2 million including 38,000 deaths^23^. As recurrent COVID-19 epidemics continue to present public health burdens, the genomic landscape of SARS-CoV-2 viruses circulating among rural populations remains understudied.

Genome sequencing has been a powerful tool for disease surveillance. Sequencing technology has been used to identify new variants and improve our understanding of their introductions and dispersions between and among states and countries, including in the United Kingdom, Brazil, and across Africa^1,24–42^. These reported studies often rely heavily on publicly available databases such as Global Initiative on Sharing Avian Influenza Data (GISAID) EpiCOV^43–45^ containing over 14 million SARS-CoV-2 genome sequences, which include limited geographic, clinical, and epidemiologic metadata. Thus, few studies have investigated the differences in SARS-CoV-2 genomic landscapes between urban and rural communities.

Prior studies on other viral diseases have illustrated that rural areas have characteristics distinct from urban and have been historical sources of infectious disease outbreaks, including mumps, influenza, and zoonotic diseases^46^. Similarly, Cuadros et al. reported that rural areas became the epicenter of the COVID-19 pandemic in 2020 with increased incidence and worse outcomes, particularly in the US Midwest^13^. Studies suggest that pathogens circulating among rural communities may have implications for virus evolution and transmission, and inclusion of these populations is crucial for disease control. To understand the role of rural populations in the evolution and transmission of SARS-CoV-2 during the early pandemic period, we collected and sequenced 979 geocoded COVID-19 respiratory specimens from Southwest Missouri representing urban and rural populations. Our study period was set between July and December 2020 to include the time when widespread diagnostic testing became available in the region and prior to the introduction of COVID-19 vaccinations, allowing us to study an overall vaccine-naïve population. Using this unique dataset from Southwest Missouri, we demonstrate that rural communities may have served an important role in the early evolution and transmission of SARS-CoV-2.

## Results

### Study population

**Fig. 1:**
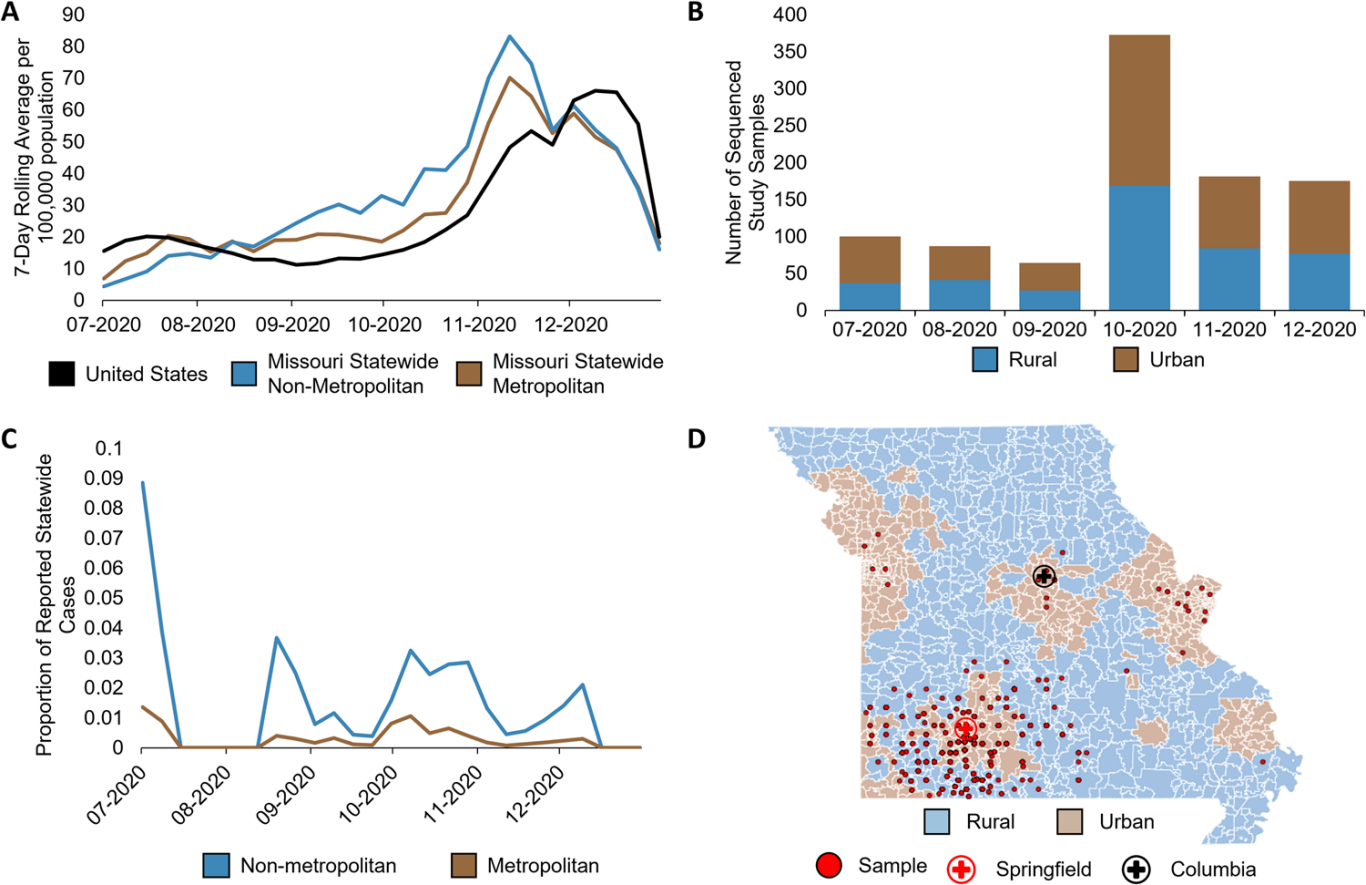
COVID-19 case and sample distribution in Missouri. **A** 7-day rolling average of statewide COVID-19 cases normalized to per 100,000 population in Missouri compared to the United States. Urban–rural designations per county was determined using US Department of Agriculture Economic Research Service Rural-Urban Continuum Codes based on Federal Information Processing Standard (FIPS) codes (https://www.ers.usda.gov/data-products/rural-urban-continuum-codes.aspx), accessed on January 23, 2022. County data were extracted from The New York Times on January 23, 2022 (https://raw.githubusercontent.com/nytimes/covid-19-data/master/rolling-averages/us-counties.csv). 7-day rolling averages were calculated based on the sum of all urban or sum of all rural cases per week divided by 7 days, divided by total Missouri urban (metropolitan) population (5,219,770) or total Missouri rural (nonmetropolitan) population (1,560,719), respectively, multiplied by 100,000. Kansas City and Joplin were reported separately by the New York Times. Kansas City lies within Jackson County and was added to the Jackson County data, whereas Joplin lies primarily within Jasper County, and thus, Joplin was added to Jasper County Data. Jackson, Jasper, and Newton counties are all considered urban counties. **B** Number of urban and rural COVID-19-positive nasopharyngeal swab samples collected for this study per month. **C** Proportion of urban and rural study samples compared to total cases within metropolitan and non-metropolitan counties, respectively, reported by the New York Times, from which samples were collected. **D** Geographic distribution of study samples.

Between July and December 2020, Missouri reported over 835,000 COVID-19-positive cases^47,48^. The statewide rolling average of cases in Missouri steadily increased from July, peaked in November with a 7-day rolling average of confirmed cases of 83.3 per 100,000 population in rural counties and 70.2 in urban counties, then gradually declined through December of 2020^47,48^. Interestingly, by August 2020, the statewide seven-day rolling average per 100,000 population of COVID-19 cases in Missouri surpassed that of the national average, and statewide per capita cases in rural areas surpassed those in urban areas, remaining predominant until December 2020 (Fig. 1A)^48^, highlighting differences in transmission dynamics between urban and rural populations within 1 year of the pandemic.

With the goal of understanding the urban–rural virus evolution and transmission dynamics across Southwest Missouri, we collected nasopharyngeal swab samples from two major medical centers across all available ZIP Codes to ensure temporospatial representation (Methods). Among our study population, 1145 positive COVID-19 samples from July to December 2020 were sequenced. Of these samples, 979 (544 associated with urban ZIP Codes and 435 with rural) contained sufficient RNA for sequencing and lineage assignment (Fig. 1B). Comparing the number of study samples sequenced to the number of reported cases by county, we sampled between 0.4 and 8.9% of all weekly reported rural cases and between 0.08 and 1.4% of reported urban cases during sampling periods (Fig. 1C). As such, our dataset encompasses representative sampling from both urban and rural communities in Southwest Missouri (Fig. 1D), allowing us to explore urban–rural dynamics.

### SARS-CoV-2 genomic diversity differed between urban and rural sequences

**Fig. 2:**
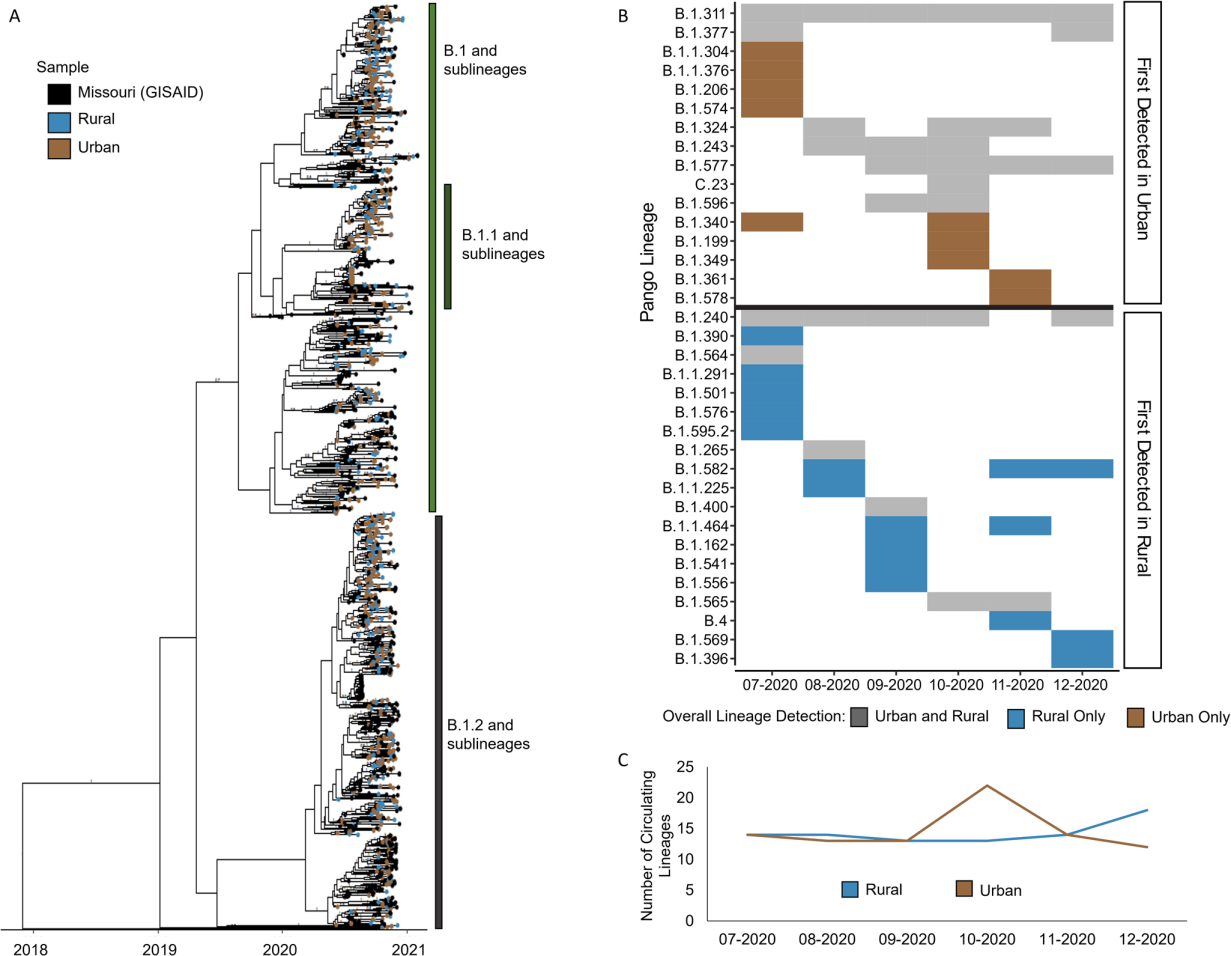
Lineage diversity within Missouri. **A** Time-scaled phylogenetic tree containing all complete public Missouri sequences from July through December 2020 from GISAID (*n* = 553) and all study sequences (*n* = 979). Branches with a posterior probability ≥0.7 and major Pango lineages are labeled. **B** Monthly detected lineages among study samples are shown by month of specimen collection and grouped by first detection in urban or rural. Colors represent detection in only urban samples (brown), only rural samples (blue), or both (gray) throughout the full study period. **C** Virus diversity calculated by the number of lineages detected in each geographic region (urban or rural) during each month.

To understand the overall genomic landscape of SARS-CoV-2 and determine the generalizability of our dataset to Missouri as a whole, we integrated our sample data of 979 sequences with the 553 publicly available complete Missouri SARS-CoV-2 sequences from GISAID during the same study period (Supplementary Table 1, Fig. 2A). Of the Pango lineages detected among all studied samples, 26 were found in both GISAID and samples from our study, representing 1421 (92.8%) of the 1532 total Missouri samples. An additional 21 lineages were identified only in the public samples (*n* = 38 samples, 2.5%), and 27 were found only in our study samples (*n* = 74 samples, 4.8%). In both study and public datasets, lineages B.1 and B.1.1 were predominant in July 2020, and by September 2020, lineage B.1.2 became predominant and remained the primary lineage through December 2020, indicating consistency of predominant circulating viruses and generalizability of our sample population.

Because urban–rural classification is unavailable for samples from the public database, our subsequent analyses on urban–rural differences utilize only samples from our study. To begin the comparison of the urban–rural genomic landscapes, we first surveyed the overall Pango lineage diversity among our urban and rural samples. A total of 53 Pango lineages were identified among our samples. Eighteen lineages were detected in both urban and rural communities within the same month whereas seven lineages were first detected among urban and five among rural before detection in the alternate urban–rural classified sample in later months (Supplementary Fig. 1). Nine lineages were exclusively detected in our urban sequences and 14 only in rural (Fig. 2B). Further, we found that the overall number of circulating lineages detected in rural areas steadily increased after October 2020 and surpassed that of urban after November 2020 (Fig. 2C). A peak in the number of detected lineages was seen in urban communities in October 2020, along with the emergence and predominance of lineages B.1.2 and B.1.234. These findings highlight the differences in circulating viruses and lineage diversity between urban and rural communities.

The number of detected circulating lineages alone does not account for samples sizes in each lineage and may bias our analyses towards low frequency lineages which are easily missed during sampling. Thus, we also analyzed viral intrapopulation genetic diversity (IGD), calculated as the mean pairwise genetic distance among sequences for each geographic group (total, rural, and urban) (Methods). The IGD for rural samples was 0.081% (standard error [SE] = 0.0067%) whereas that for urban samples was 0.0844% (SE = 0.0067%) during the study period. The overall IGD among all study samples was 0.0830% (SE = 0.0069%). Comparison of IGD between urban and rural samples showed that overall urban IGD was significantly higher than rural IGD (Wilcoxon test, *p*-value < 0.001) (Supplementary Fig. 2). Thus, while we saw an increase in circulating viruses among our rural samples, we saw higher intrapopulation genetic diversity among urban samples, suggesting that the circulating viruses in rural were predominated by a few lineages.

Taken together, the predominant lineages appeared in both urban and rural areas. We detected a spike in the number of circulating lineages among urban samples in October 2020 before decreasing in November 2020. On the other hand, we detected an increase in lineage diversity among rural samples from October to December 2020 and multiple lineages that were only detected among our rural samples. Further analyses of pairwise genetic distances revealed that urban samples had overall greater genetic diversity than rural, suggesting that the diverse lineages detected in rural appeared in lower frequencies compared to the predominant lineages.

### SARS-CoV-2 diffusion involved bi-directional transmission links

Next, we investigated the question of SARS-CoV-2 spread between urban and rural communities. To do this, we used phylogeographic approaches to analyze the eight largest Pango lineages, each of which included at least 10 urban and 10 rural sequences (B.1, B.1.1, B.1.1.337, B.1.2, B.1.234, B.1.240, B.1.311, and B.1.509). All available samples in these study lineages were included in our analyses. To ensure the robustness of our study, we included genetically close sequences available in the GISAID public database, including both Missouri and non-Missouri sequences (Methods). For each lineage, we analyzed for transmission links between urban and rural communities as well as potential transmission links with publicly available samples.

**Fig. 3:**
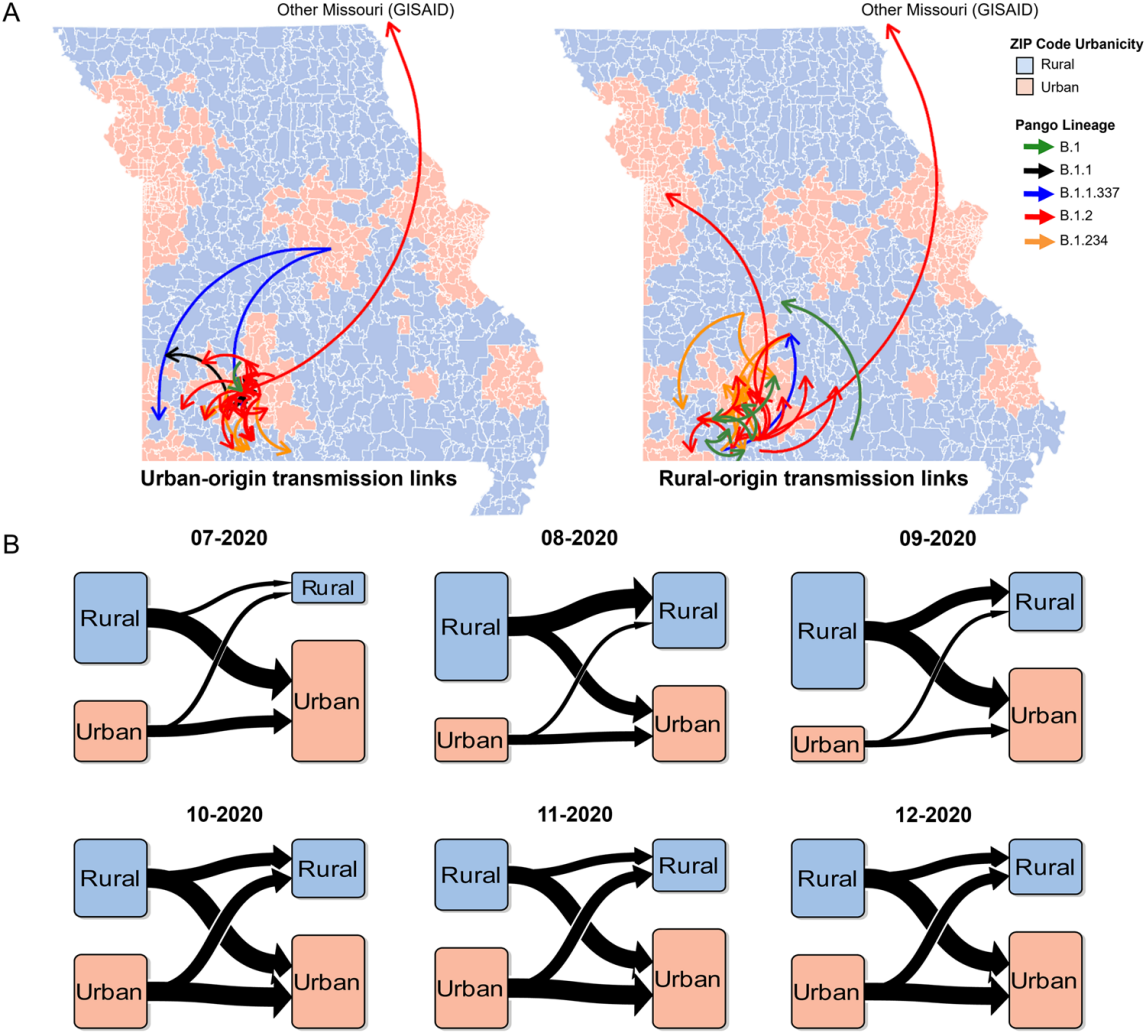
Transmission links across Southwest Missouri. **A** Maps of transmission links for each lineage containing at least 10 urban and 10 rural samples that are urban-origin (left) and rural-origin (right). Arrows represent transmission links defined as Bayes factor ≥3 and posterior probability ≥0.7, and arrowheads point towards the direction of transmission. Bayesian Stochastic Search Variable Selection (BSSVS) was used for phylogeographic analyses. **B** Transmission origins by geographical category for each lineage. Arrow widths represent relative proportion of transmission links compared to the monthly total transmission links.

Based on geographic location resolution, we categorized these sequences into four groups: urban (*n* = 529), rural (*n* = 426), Missouri GISAID (*n* = 56), or Not Missouri GISAID (*n* = 618). A total of 92 transmission links were detected between July and December 2020 (Supplementary Table 2). The transmission links originating in urban areas constituted 34% (*n* = 31) of the total, and they were associated with lineages B.1, B.1.1, B.1.1.337, B.1.234, and B.1.2. Transmission links originating in rural areas accounted for 33% (*n* = 30) and were associated with lineages B.1, B.1.1.337, B.1.234, and B.1.2 (Fig. 3A). Multiple highly probable diffusions were observed bidirectionally from both rural to urban (17 transmission links) and urban to rural (11 transmission links) communities (Supplementary Fig. 3). The remaining transmission links originated from Missouri GISAID samples of unknown urban–rural classification (*n* = 4) or from Not Missouri GISAID samples (*n* = 27).

To elucidate the temporal dynamics in diffusion events between urban and rural areas, we analyzed the relative number of urban–rural transmission links by month (Fig. 3B). Monthly rural-origin transmission links were more frequent than urban from July through September 2020, whereas monthly urban-origin transmission links became more prevalent beginning in October 2020 and remained elevated through December 2020. Of interest, the frequency of rural to urban transmission links consistently outpaced urban to rural transmission. Overall, our analyses demonstrated high probability for bi-directional diffusions between rural and urban sequences.

### Rural SARS-CoV-2 lineages spread locally and globally

The design of our unique urban–rural dataset also allowed us to explore SARS-CoV-2 evolutionary differences in rural versus urban settings. We started by exploring whether new lineages may have emerged in rural communities and consequently spread to urban or even outside Missouri. We generated time-scaled phylogenetic trees for each of the eight lineages included in the aforementioned phylogeographic analyses. Results showed at least seven lineages that were first detected from rural communities (Supplementary Fig. 4). Among them, three rural lineages appeared to transiently circulate in Missouri, whereas the other four were associated with later sequences from beyond Missouri both nationally and globally.

**Fig. 4:**
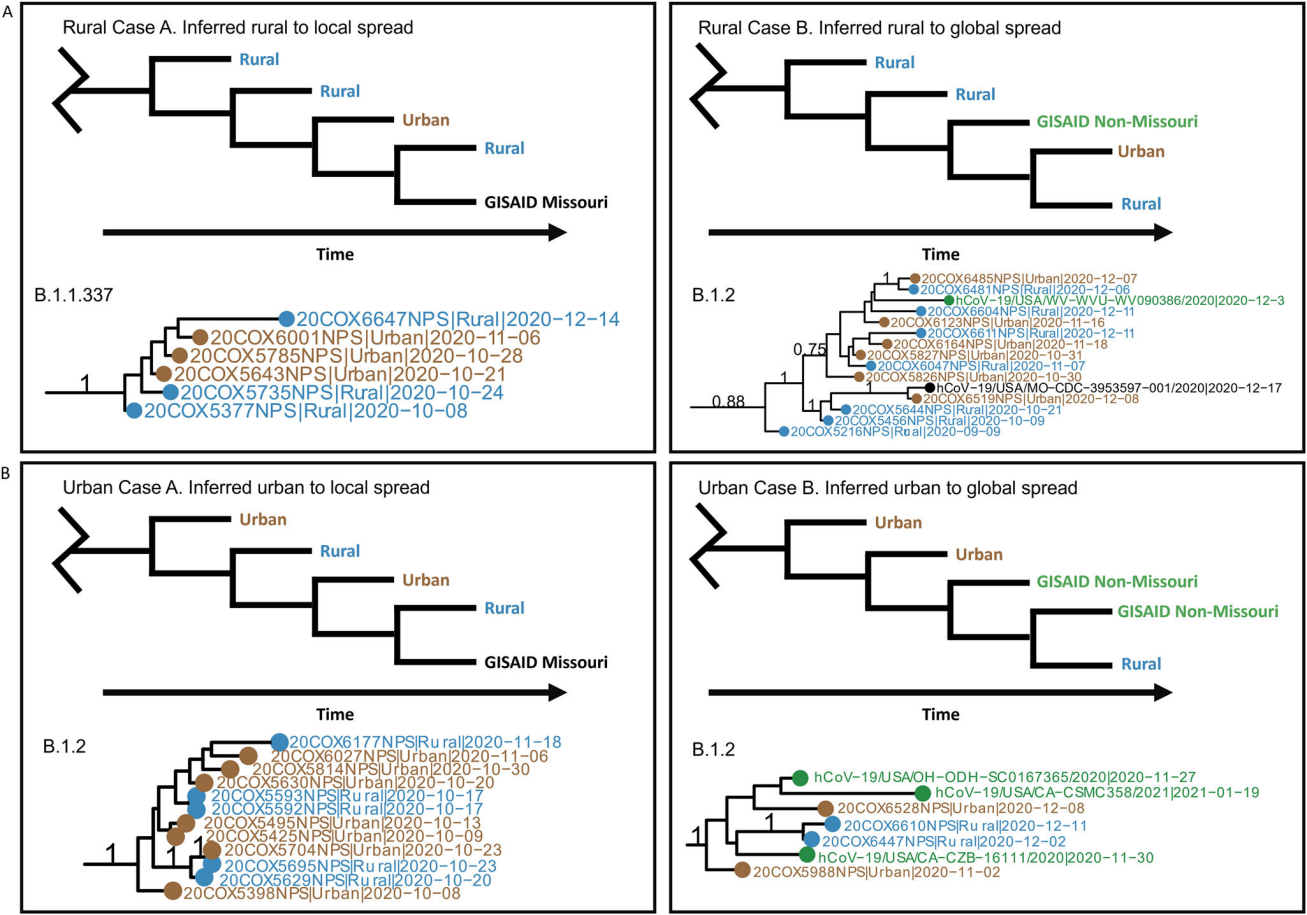
Scenarios of lineage emergence and spread from urban and rural communities. Multiple lineages were identified to have emerged from rural (**A**) and urban (**B**) sequences and spread locally, defined as rural, urban, or GISAID Missouri samples (Case A) and globally, defined as outside of Missouri (Case B). For each case, an example diagram of a lineage from a time-scaled phylogenetic tree is shown along with a real-world example of occurrences of these cases. Branch posterior probabilities of >0.7 are annotated. Blue sequences indicate rural samples, brown indicates urban samples, black indicates GISAID Missouri samples of unknown urban rural classification, and green indicates GISAID non-Missouri Samples of unknown urban–rural classification. Lineage emergence was identified by adapting the Pango lineage criteria^102^: (1) sequences share a single common ancestor; (2) the clade contains at least five sequences; (3) the clade includes at least one internal node. Further, we defined rural lineages as the largest monophyletic clade with a posterior probability of >0.70 and a single first ingroup branch involving a rural sample with the earliest date among the clade. Urban lineages are likewise defined (Methods). Full phylogenetic trees are available in Supplementary Fig. 4.

In comparison, at least nine of the newly identified lineages were inferred to have emerged from urban communities (Supplementary Fig. 4), three of which appeared to circulate in Missouri, and five were associated with later sequences outside of Missouri. Examples of these rural-emergent and urban-emergent cases are illustrated in Fig. 4A, B, respectively.

We next inferred the duration of detection and time for the emergence of a new lineage to spread using the sample collection dates. Rural-origin lineages had an average of a 72-day duration (SD = 26.2) and urban-origin lineages had an average of a 70-day duration (SD = 27.4). On average, it took 41.3 days (SD = 22.1) for a rural-origin lineage to be detected in urban areas, while an urban-origin lineage was detected in rural areas in only 22.1 days (SD = 16.6). Furthermore, rural-origin and urban-origin lineages took 83.8 days (SD = 45.5) and 66.4 days (SD = 20.8), respectively, to spread beyond the borders of Missouri (Supplementary Fig. 5).

**Fig. 5:**
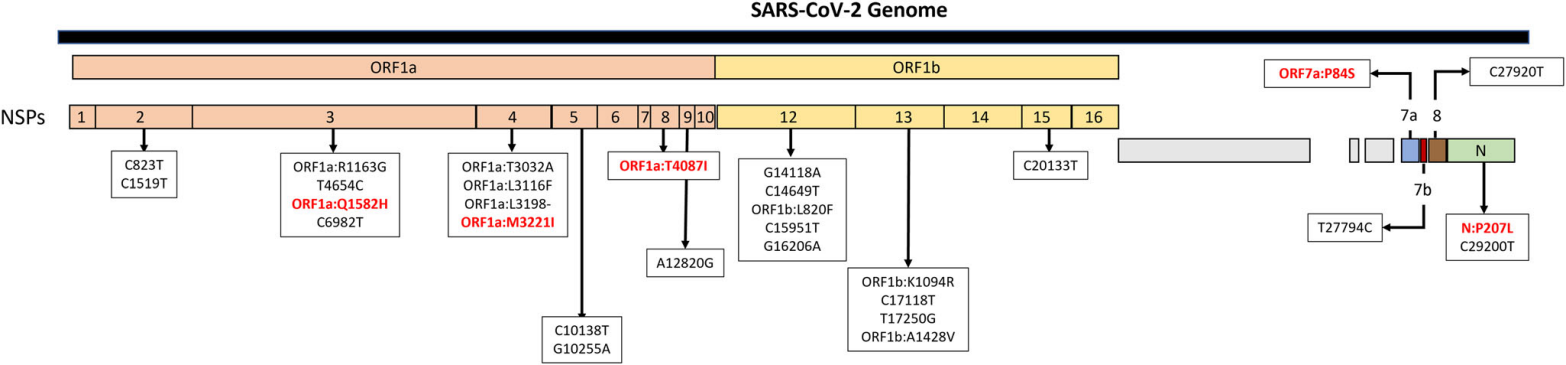
Mutations associated with Missouri-origin lineages. Representation of all mutations associated with urban-origin or rural-origin lineages across the SARS-CoV-2 genome. Red text represents substitutions at positively selected sites; NSP non-structural protein, ORF open reading frame.

Additional molecular analyses of these 15 new Missouri lineages revealed 29 mutations, 12 of which were associated with amino acid substitutions on nonstructural protein (NSP)3, NSP4, NSP8, NSP12, NSP13, and open reading frame (ORF)7a (Fig. 5, Supplementary Fig. 4). Substitutions ORF1a:Q1582H, ORF1a:M3221I, ORF1a:T4087I, ORF7a:P84S, and nucleocapsid(N):P207L were also undergoing positive selection (Supplementary Table 3 and Fig. 5).

In summary, our findings indicate that new lineages frequently emerged in both urban and rural areas of Missouri. These new lineages independently acquired a number of mutations, some of which were also detected in other lineages. While both rural-origin and urban-origin lineages had the potential to spread beyond Missouri, the former exhibited a slower rate of spread.

### N:R203K/G204R substitutions were associated with urban–rural classification and positive selection

**Fig. 6:**
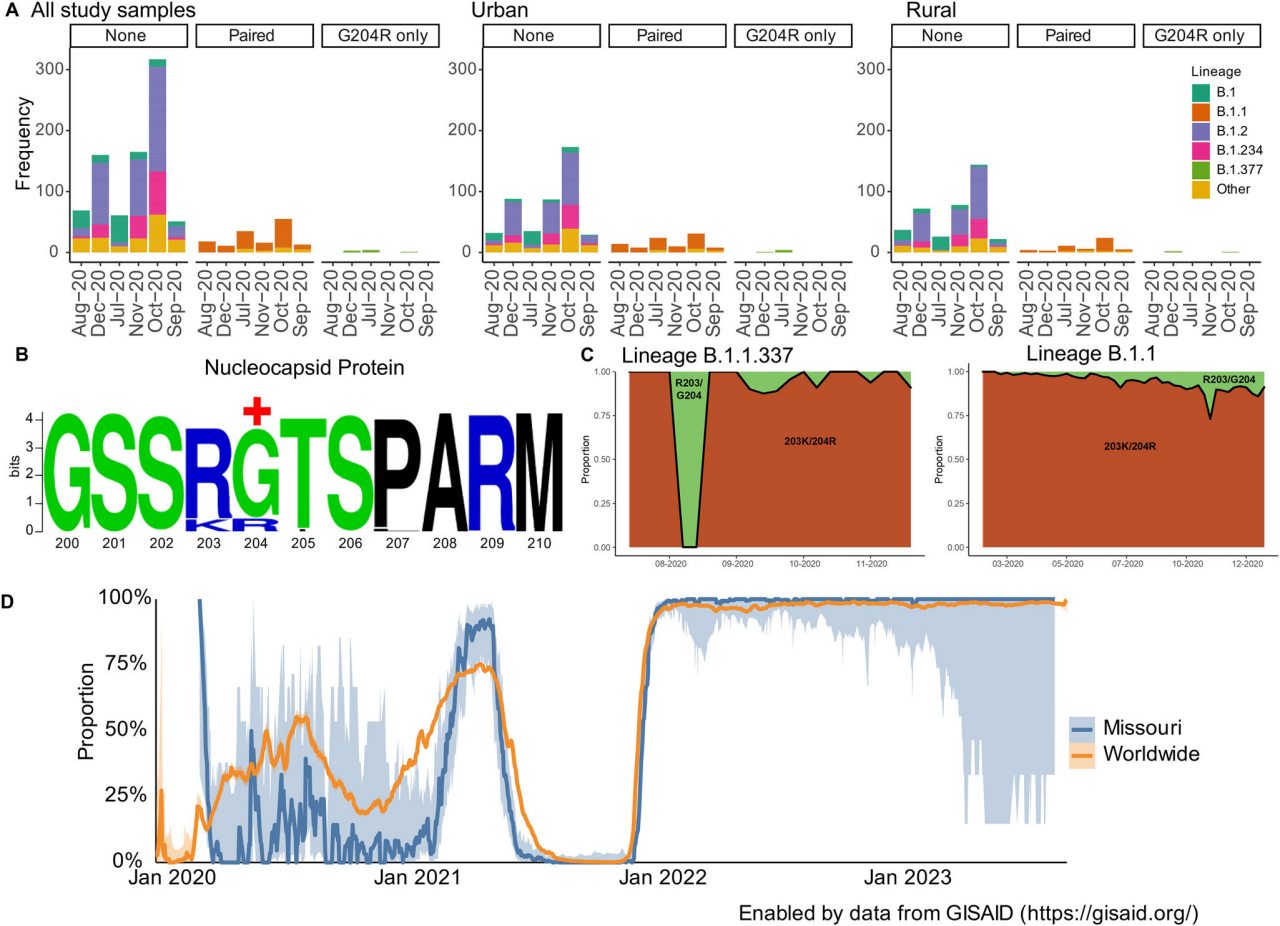
Association of substitutions N:R203K/G204R with urban–rural classification. **A** Monthly frequencies of study samples containing no substitutions, both substitutions, and a single substitution (N:G204R) at residues 203 and 204 along the nucleocapsid protein. Colors represent the lineages occurring in at least 10% of the study population. All other lineages were labeled as “Other.” **B** Pervasive positive selection was detected at residue 204 on the nucleocapsid protein among rural (shown), but not urban sequences. Sequence logo was generated using https://weblogo.berkeley.edu/. +, pervasive positive selection detected using HyPhy. **C** Weekly proportion of sequences containing the N:R203K/G204R substitutions (orange) compared to those without (green) among B.1.1.337 and B.1.1 lineage viruses. Data were extracted from outbreak.info (https://outbreak-info.github.io/R-outbreak-info/). **D** Comparison of the proportion of GISAID sequences containing both N:R203K and N:G204R substitutions globally and in Missouri with 95% confidence intervals. Proportions were calculated using the 7-day rolling average of samples containing these substitutions per total rolling count. Prevalence data was extracted from and plotted using outbreak.info (https://outbreak-info.github.io/R-outbreak-info/).

Finally, our unique dataset allowed us to explore urban–rural evolutionary differences at the amino acid level. We first analyzed all amino acid substitutions occurring at a minimum of 10% frequency (20 amino acid substitutions) among our study samples. Of interest, we found that urban–rural classification was associated with only the paired substitutions, N:R203K/G204R, which emerged during the early pandemic period and later became predominant with multiple past and present variants of concern (VOCs). Our study samples have a mixture of patterns at these two residues: N:R203/G204 (*n* = 823), N:R203/R204 (*n* = 8), and N:K203/R204 (*n* = 148). Both substitutions R203K and G204R were more predominant with urban samples compared to rural (N:R203K, *p*-value = 0.029; N:G204R, *p*-value = 0.032) (Fig. 6A).

The B.1.377 lineage contained the codon AGA, resulting in amino acid substitute N:G204R (but not N:R203K). In contrast, at the same positions, B.1.1 and its sub-lineages, including a proportion of B.1.1.337 sequences, harbored codon CGA, resulting in N:R203K/G204R (Supplementary Fig. 5). Positive selection analyses revealed that N:204 was undergoing pervasive positive selection among rural samples (posterior probability > 0.90), particularly those associated with B.1.377 lineage, but not in urban samples (Fig. 6B).

Further exploration of these co-occurring mutations among publicly available data demonstrates that lineage B.1.1 was first detected globally in February 2020 and N:R203K/G204R was present in 40,515 of 42,272 sequences as of December 2020, and sublineage B.1.1.337 was first detected globally in August 2020 with N:R203K/G204R in 184 of 191 sequences as of December 2020 (Fig. 6C). The patterns of N:R203K/G204R prevalence was consistent between publicly available global and publicly available Missouri sequences and were detected in the vast majority of all cases throughout 2022 (Fig. 6D). In contrast, lineage B.1.377, seen in our rural samples, was only detected globally between April 2020 and May 2021 in 280 total sequences submitted to GISAID^49^. While B.1.377, the lineage driving the positive selection pressure in our rural samples at position N:204, was circulating at low levels and is no longer detected, the global predominance of the paired substitutions at N:203 and N:204 imply fitness advantage at these positions.

Overall, these results show that samples from urban and rural communities had differing patterns and selection pressures at positions 203 and 204 on the nucleocapsid protein. Although the prevalence of these substitutions was higher in urban communities, residue 204 on the nucleocapsid protein was undergoing positive selection in association with the single G204R substitution in lineage B.1.377.

## Discussion

From its emergence in 2019, SARS-CoV-2 quickly evolved into multiple variants that differed phenotypically^50–52^. Most major variants were first identified in large urban centers with robust surveillance capabilities^53–57^. However, the geographic origins of these variants remain unclear^58^, challenging prevention and control efforts. Determining the origins of emergence remains elusive due to biased sampling and subsequent surveillance and analyses from large urban centers^51,59–64^ and the lack of granular geographic metadata associated with publicly available sequences. Consequently, by the time new viruses are detected, transmission has likely already begun, as seen with the detection of the Omicron variant in South Africa^58,65^. Thus, understanding the evolution and transmission dynamics of emerging SARS-CoV-2 variants, particularly among largely understudied rural populations, is essential for facilitating the timely development of regionally relevant public health and surveillance strategies^66^. Our study utilizes a unique dataset of geocoded samples from Southwest Missouri that allowed for specific analyses of urban versus rural events at a more granular level than most studies and databases. This made it possible for us to identify transmission events and infer directionality while also comparing evolutionary dynamics between urban and rural samples. Overall, we demonstrated that rural communities serve an important role in the diversity, spread, and evolution of SARS-CoV-2.

While published literature is limited regarding the emergence and spread of genetic variants, the unique dataset curated for this study advances our knowledge of SARS-CoV-2 among rural communities by identifying numerous probable bi-directional transmission links, particularly from rural to urban. This is consistent with an epidemiological model constructed by Polo et al. which further supports transmission occurring at the urban–rural interface^42^. Additionally, seroprevalence studies have shown higher than expected COVID-19 infections among rural populations during the early pandemic^67^ and increased infections rates in rural communities compared to urban during the second wave (November 2020–March 2021) of the pandemic^22^, consistent with our findings from genomic analyses. An early study by Paul et al. reported an increase of mean prevalence of COVID-19 from 3.6 to 43.6 per 100,000 population within three weeks in April 2020 among rural counties in the United States^68^, further demonstrating the rapid transmission of COVID-19 in rural communities. Rural virus spread has been attributed to community contacts and “super-spreader” events allowing opportunities for high viral replication and consequently, increased opportunities for substitutions and recombination events^69–76^. Additionally, rural to urban spread may have occurred during commutes to healthcare facilities, work, and shopping and entertainment centers and during holiday travel. Preventative measures, specifically aimed at the urban–rural interface, may be beneficial towards controlling transmission between these communities, and public health guidelines and mitigation strategies should be tailored towards the timely needs of individual populations.

Our results also demonstrated that rural populations may be an important source of new lineages with potential to become transmitted globally. Phylogenetic analyses revealed seven lineages that were first detected among our rural samples, four of which were associated with later samples detected both nationally and globally. Many of these lineages also contained substitutions at positively selected sites. Further analyses of amino acid substitutions revealed two amino acid substitutions, N:R203K and N:G204R, that were associated with urban–rural classification. Compared to N:R203/G204 in the original pandemic virus, different combinations of substitutions such as N:R203/G204, N:R203/R204, and N:M203/G204 have been reported (Supplementary Table 4). The paired N:R203K/G204R substitutions were first detected globally on January 4, 2020 and predominated in the Alpha, Gamma, and Omicron variants whereas a single substitution, N:R203M, was associated with the Delta variant^49^. These patterns suggest multiple independent parallel emergences of these substitutions and convergent evolution, which is supported by prior studies demonstrating increased fitness advantage^77–80^ and findings of positive selection at these sites. Our results further showed positive selection at N:204 for rural but not urban sequences during 2020 which appeared to be driven by sequences from lineage B.1.377, suggesting that viruses circulating among rural areas may influence evolutionary pressures.

Despite our expansive sampling using temporospatial criteria, our samples are biased toward individuals who actively sought out testing. Additionally, phylogenetic topology is largely dependent on sampling and should only be used to approximate virus epidemiology. Our sampling area was limited primarily to Southwest Missouri, which would not account for variants that may have emerged from unsampled regions such as the neighboring urban centers Kansas City or Saint Louis and introduced to our study population. We included publicly available samples in our analyses to account for this bias. Additionally, the consistency of our predominant lineages in comparison to those seen publicly suggest generalizability for our findings. Additionally, the demography of rural communities in Missouri (93% White, 19.4% aged 65 years and above, 13.7% living below the federal poverty line, and 14.6% without health insurance)^81^, is representative of the rural populations across the nation (89% White, 18% aged 65 years and above, 18% living in poverty^82^, and 16% without health insurance)^83^, further suggesting the representative nature of our study population and findings for other rural communities. Finally, additional studies are necessary to understand the implications of COVID-19 vaccines, which have a lower uptake in rural than urban communities, and VOCs, which have been shown to increase virus infectivity^71,84–88^, transmissibility^1–3^, and evasion of host immunity^4–8^ on rural SARS-CoV-2 evolution and transmission.

In summary, we investigated the genome landscapes of urban and rural populations in Southwest Missouri and demonstrated that rural communities likely played a critical role in SARS-CoV-2 evolution and transmission during an early vaccine-naïve pandemic period and cannot be ignored when developing public health guidelines. In addition to increased public health measures that are relevant to the local and rural populations and at urban–rural interfaces, resources for genomic surveillance should be allocated to allow for active and rigorous inclusion of rural populations. These efforts can help to identify sources of variant emergence and spread more quickly and allow for regionally relevant interventions. While much more work is needed to determine the different sources and transmission routes of SARS-CoV-2 variants, this study furthers our understanding of these concepts by highlighting the essential role of rural communities in the emergence and spread of new SARS-CoV-2 variants.

## Materials and methods

### Ethical approval

This study has been approved by University of Missouri Institutional Review Board (IRB #2025449, #2049364).

### Sample selection and diagnosis

COVID-19-positive nasopharyngeal swabs were collected with associated ZIP Codes between July 1, 2020–December 31, 2020 from CoxHealth in Springfield, Missouri, a major Missouri healthcare system located in Springfield, Missouri that serves 25 counties throughout Southwest Missouri and Arkansas, and University of Missouri Health Care in Columbia, Missouri, serving another 25 counties. Samples were tested for diagnostic purposes using the Centers for Disease Control (CDC) 2019 Novel Coronavirus Real-Time Reverse Transcriptase (RT)-PCR Diagnostic Panel, and positive samples were collected to encompass each week of the pandemic and all available ZIP Codes. For genome sequencing, samples were further randomly selected after stratifying for time and location targeting three samples per ZIP Code per week, to optimize representative sampling of all available weeks and ZIP Codes.

### Urban–rural classification

Due to our interest in urban–rural transmission of SARS-CoV-2, we utilized urban–rural designations at the ZIP Code level. We determined the urban and rural category of each individual using Rural–Urban Commuting Area (RUCA) codes as defined by the United States (US) Department of Agriculture Economic Research Service^89^. These RUCA codes are defined by population density, urbanization, and commuting patterns, allowing us to conduct unique analyses that captures the minutia of genome variation and spread among urban and rural communities. We further classified these ZIP Codes as either urban or rural in consistency with the dichotomous categories defined by the University of Washington Rural Health Research Center^90^.

### Genetic sequencing and assembly

SARS-CoV-2 whole genome RT-PCR amplification was conducted using the Juno system (Fluidigm Corporation, CA, USA)^91^ with 47 pairs of custom designed specific primers targeting the reference sequence Wuhan-Hu-1 (Accession Number: NC_045512.2). Amplicon libraries were then prepared using the Illumina DNA Prep kit, followed by sequencing with either MiSeq Reagent Kit v3 (600-cycle) and MiSeq sequencing system or NovaSeq Reagent Kit SP v1.5 (300-cycle) on NovaSeq 6000 instrument (Illumina, San Diego, California, USA).

The quality of paired-end reads obtained from MiSeq sequencing were analyzed and consensus sequences were constructed using Qiagen CLC Genomics Workbench 20.0.4. Sequences were imported as paired reads and trimmed with a quality score of 0.05. The trimmed reads were mapped to the reference genome, Wuhan-Hu-1 (Accession Number: NC_045512.2). A minimum coverage depth of 10 was required for assembling the consensus sequences.

### Genetic diversity calculations

Viral genetic diversity analyses were performed using MEGA-CC (Molecular Evolutionary Genetics Analysis Compute Core) v10.1.8^92^. To estimate the mean evolutionary diversity for the entire population, rural population, and urban population, we used a Tajima-Nei model^93^ with 1000 bootstrap replications. The 1st, 2nd, 3rd, and noncoding codon positions were included, and all ambiguous positions were removed for each sequence pair (pairwise deletion option). We further assessed interpopulation diversity by comparing the pairwise genetic distance distributions between urban and rural samples. Estimation of interpopulation genetic diversity was calculated using the pairwise distance among sequence pairs between urban and rural populations in total and by month. The maximum composite likelihood model was applied with 1,000 bootstrap replications, a ⌈_4_ rate variation, and a partial deletion option that removed positions with <50% site coverage^94^. These results were summarized and visualized using Rstudio 2022.07.2 and the following R packages: rio, tidyverse, readr, graphics, sm, gridExtra, ggplot2, ggpubr. Statistical significance was analyzed with a Wilcoxon signed-rank test and a *p*-value < 0.05.

### Phylogenetic analyses

The samples downloaded from GISAID were filtered to include only samples collected through December 31, 2020 to overlap with our sampling period and analyze spread. Of 9,319,311 samples available on March 16, 2022, 619,560 samples remained after filtering for quality and collection date. Due to the large scale of sequences, an alignment-free approach was used to identify the samples from the public databases with the closest distance from our samples. We used a complete composition vector (CCV) algorithm^95^ to generate distance matrices among our samples and those in the GISAID database. The top three matches for each sample were extracted, and duplicates were removed. Additionally, all publicly available Missouri sequences from GISAID were included in the analyses. Sequences were aligned using Nextclade^96^.

Time-scaled phylogenetic trees were generated for each lineage with at least 10 rural study samples and 10 urban study samples for a total of eight lineages. Phylogenetic analyses were performed using BEAST v2.6.7. The Hasegawa, Kishino, and Yano (HKY) + ⌈_4_ substitution model with an exponential coalescent growth prior was applied to allow for variable base frequencies and a strict molecular clock was used^97,98^. We performed independent runs with chain lengths of 50,000,000 steps and sampled every 5,000 steps, resulting in 10,000 samples per run for each tree. A burn-in of 10% was used to remove initial steps that represented poor configuration. Time-scaled trees were summarized with TreeAnnotator and visualized using the R package “ggtree,” and a posterior probabilities cutoff of 0.70 was used^99^ to assess the confidence of tree topology^31,32,100^.

Due to the size of Pango lineage B.1.2, we first generated a maximum likelihood tree using FastTree v2.1.11^101^ for an estimated tree topography, then separated the samples into six subsets based on the clades estimated by the maximum likelihood tree to construct the Bayesian trees for computational feasibility. All trees were rooted to hCoV-19/Wuhan/PBCAMS-WH-01/2019 (EPI_ISL_402123; 2019-12-24). The following reference sequences were also included in each tree to create an outgroup: hCoV-19/Germany/BW-ChVir-1577/2020, hCoV-19/Australia/VIC273/2020, hCoV-19/Germany/BY-ChVir-929/2020, and hCoV-19/USA/WY-WYPHL-20086942/2020.

### Lineage classification

The Phylogenetic Assignment of Named Global Outbreak Lineages (PANGOLIN) software was used (PANGO v4.0.6 (2022-04-22)) to classify Pango lineages for each sample^102^. Sequences with a genomic coverage of >50% was sufficient for lineage determination and were included in the subsequent analyses. All sequences were submitted to GISAID.

To identify rural and urban Missouri lineages that are not yet recognized by PANGOLIN, time-scaled phylogenetic trees were generated for each lineage with at least 10 urban and 10 rural samples described above (Supplementary Fig. 2). Novel variants were identified by adapting the PANGOLIN lineage criteria^102^: (1) The sequences share a single common ancestor and represent a monophyletic or paraphyletic clade; (2) the clade contains at least five sequences; (3) the clade includes at least one internal node consistent with onward transmission. Further, we defined rural lineages as the largest monophyletic clade with a posterior probability of at least 0.70 and a single first ingroup branch involving a rural sample with the earliest date among the clade. Urban lineages are likewise defined using a single first ingroup branch involving an urban sample with the earliest date among the clade.

### Amino acid substitution and positive selection analysis

To determine whether urban–rural classification was associated with amino acid substitutions, we filtered all sequences for those with ≥ 95% genome coverage. Mutations were extracted using Nextclade^96^ and summarized and analyzed in Rstudio. Amino acid substitutions were further filtered for those occurring at least 10% among the study population. Fisher exact tests were used to assess each remaining mutation. A *p*-value < 0.05 was considered significant.

Positive selection analysis was performed on all study sequences without ambiguous bases along each gene and each SARS-CoV-2 gene was analyzed separately. Maximum likelihood newick trees were generated using Molecular Evolutionary Genetics Analysis (MEGA) X v10.1.8 with 1000 bootstrap replications. The Hasegawa, Kishino, and Yano (HKY)+gamma (⌈) (using 4 gamma distributed rate categories) substitution model was used in consistency with phylogenetic and phylogeographic analyses. Nearest-Neighbor-Interchange was used as the maximum likelihood heuristic method. The Genetic Algorithms for Recombination Detection (GARD) v0.2 method^103^ (General Discrete model rate variation and 3 rate classes) was used on datamonkey.org to screen sequences of each gene for recombination. No recombination was detected. Subsequently, selective pressure was analyzed using the FUBAR (Fast, Unconstrained Bayesian AppRoximation) v2.2 method from the HyPhy software v2.5.42(MP)^104,105^. Significance was defined at sites with a posterior probability >0.9, which is strongly suggestive of positive selection^104^.

### Phylogeographic analysis

To identify transmission links between urban and rural areas in Missouri, phylogeographic analyses were conducted for the lineages analyzed for the phylogenies above using BEASTv1.10.4 and Bayesian modeling with the parameters selected during the phylogenetic analyses^106^. Using the network inferred by the Bayesian Stochastic Search Variable Selection (BSSVS) procedure, we identified highly probable transmission links as those with a posterior probability of ≥0.70 and Bayes factor (BF) ≥ 30 to demonstrate statistical evidence for a transmission event^107^. ZIP Codes, labeled with their urban–rural designations, were utilized as discrete variables. Geographical coordinates were further added for the visualization of urban–rural SARS-CoV-2 migrations, and transmission events were extracted using SpreaD3^108^. Other Missouri sequences that were publicly available in GISAID were assigned a ZIP Code of 11111, and out-of-state sequences from GISAID were assigned a ZIP Code of 22222 to consolidate the results. Transmission links were further visualized using the R package “ggplot” v.3.3.2^109^. The summary of transmission links shown in Fig. 3B were generated using the R package “DiagrammeR” v0.8.4 (https://rich-iannone.github.io/DiagrammeR/).

## Supplementary information

Supplemental Materials Submit

## Data Availability

All publicly available sequences and associated metadata used in this dataset are published in GISAID’s EpiCoV database. To view the contributors of each individual sequence with details such as accession number, virus name, collection date, originating lab and submitting lab, and the list of authors, please visit the doi listed with each dataset: *Data availability for GISAID samples included in our analyses*: GISAID Identifier: EPI_SET_220804ys (10.55876/gis8.220804ys). EPI_SET_220804ys is composed of 365 individual genome sequences. The collection dates range from 2019-12-30 to 2021-12-25; Data were collected in 42 countries and territories. *Data availability for GISAID Missouri samples utilized in our analyses*: EPI_SET ID:EPI_SET_220816fa (10.55876/gis8.220816fa). EPI_SET_220816fa is composed of 553 individual genome sequences. The collection dates range from 2020-07-06 to 2020-12-31. Data were collected in 1 country and territory. Data for study samples with >50% genome coverage are available on GISAID: EPI_SET ID:EPI_SET_231117kt doi: (https://doi.org/10.55876/gis8.231117kt).
