# Supplementary material for: Rural populations facilitated early SARS-CoV-2 evolution and transmission in Missouri, USA
Source: Npj Viruses. Author manuscript; Available in PMC 2024 Jan 5. (PMC10769004; doi:10.1038/s44298-023-00005-1)
Supplement: Supplementary Materials [file NIHMS1952328-supplement-Supplementary_Materials.pdf]

Supplementary Tables

Supplementary Table 1. Comparison of PANGOLIN lineages between study samples and publicly available samples, July-December, 2020. The Phylogenetic Assignment of Named Global Outbreak Lineages (PANGOLIN) software was used (PANGO v4.0.6 (2022-04-22)) to classify Pango lineages for each sample.

PANGO Lineage	GISAID MO	Study Samples	Grand Total
B.1	21	66	87
B.1.1	35	52	87
B.1.1.135	1	4	5
B.1.1.222	1	0	1
B.1.1.225	0	1	1
B.1.1.265	2	0	2
B.1.1.291	1	1	2
B.1.1.304	1	1	2
B.1.1.312	1	0	1
B.1.1.316	1	0	1
B.1.1.329	3	0	3
B.1.1.33	0	5	5
B.1.1.335	1	0	1
B.1.1.337	8	78	86
B.1.1.376	2	1	3
B.1.1.416	1	0	1
B.1.1.432	0	4	4
B.1.1.464	0	2	2
B.1.1.8	1	0	1
B.1.110.3	0	3	3
B.1.119	1	0	1
B.1.126	1	0	1
B.1.139	3	7	10
B.1.162	0	1	1
B.1.188	1	0	1
B.1.199	0	1	1
B.1.2	278	406	684
B.1.206	0	1	1
B.1.232	1	0	1
B.1.234	27	137	164
B.1.240	14	45	59
B.1.243	1	4	5
B.1.265	2	3	5
B.1.267	9	0	9
B.1.311	51	25	76
B.1.324	0	11	11

B.1.340	0	2	2
B.1.349	0	1	1
B.1.361	0	1	1
B.1.369	14	6	20
B.1.377	11	8	19
B.1.382	1	0	1
B.1.385	1	0	1
B.1.390	0	2	2
B.1.396	0	1	1
B.1.400	0	6	6
B.1.413	0	5	5
B.1.429	1	0	1
B.1.501	0	1	1
B.1.507	1	0	1
B.1.509	6	29	35
B.1.541	0	1	1
B.1.544	0	5	5
B.1.556	0	1	1
B.1.564	2	2	4
B.1.565	8	5	13
B.1.568	0	2	2
B.1.569	0	2	2
B.1.570	1	0	1
B.1.574	2	1	3
B.1.576	10	1	11
B.1.577	0	8	8
B.1.578	0	1	1
B.1.582	2	3	5
B.1.591	2	4	6
B.1.595	10	11	21
B.1.595.1	3	0	3
B.1.595.2	0	1	1
B.1.596	1	3	4
B.1.599	3	0	3
B.1.609	2	2	4
B.1.612	3	0	3
B.4	0	1	1
C.23	0	4	4

Supplementary Table 2. Significant transmission links across Southwest Missouri. Significance was defined as posterior probability ≥ 0.70 and Bayes factor ≥ 3.

Lineage	From	To	Bayes factor	Posterior probability	Transmission link	Transmission origin
B.1.1.337	65102-Urban	65714-Urban	157.080	0.754	Urban to Urban	Urban origin
B.1.1.337	65102-Urban	64850-Rural	241.331	0.825	Urban to Rural	Urban origin
B.1.1.337	Not MO	65102-Urban	981.755	0.950	Not MO to Urban	Not MO origin
B.1.1.337	65747-Rural	65536-Rural	137.177	0.728	Rural to Rural	Rural origin
B.1.1.337	Not MO	65738-Urban	408.176	0.888	Not MO to Urban	Not MO origin
B.1.1.337	Not MO	65807-Urban	219.534	0.811	Not MO to Urban	Not MO origin
B.1.1	65705-Urban	65810-Urban	538.342	0.943	Urban to Urban	Urban origin
B.1.1	65610-Urban	64759-Rural	138.892	0.811	Urban to Rural	Urban origin
B.1.1	Not MO	65608-Urban	29070.486	1.000	Not MO to Urban	Not MO origin
B.1.1	Other MO	65616-Rural	80.940	0.715	Other MO to Rural	Other MO origin
B.1.1	65810-Urban	65803-Urban	118.491	0.786	Urban to Urban	Urban origin
B.1.1	65810-Urban	65806-Urban	93.143	0.743	Urban to Urban	Urban origin
B.1.1	65810-Urban	65807-Urban	189.858	0.855	Urban to Urban	Urban origin
B.1.234	65536-Rural	65653-Rural	2879.260	0.976	Rural to Rural	Rural origin
B.1.234	65536-Rural	65672-Rural	188.110	0.731	Rural to Rural	Rural origin
B.1.234	65536-Rural	65742-Urban	32364.895	0.998	Rural to Urban	Rural origin
B.1.234	65631-Urban	65769-Rural	32364.895	1.000	Urban to Rural	Urban origin
B.1.234	65668-Rural	65706-Urban	667.837	0.906	Rural to Urban	Rural origin
B.1.234	65668-Rural	65723-Rural	6417.536	0.989	Rural to Rural	Rural origin
B.1.234	65669-Urban	65672-Rural	192.262	0.735	Urban to Rural	Urban origin
B.1.234	65708-Rural	65802-Urban	6417.536	0.989	Rural to Urban	Rural origin
B.1.234	65708-Rural	65803-Urban	279.451	0.801	Rural to Urban	Rural origin
B.1.234	65708-Rural	65807-Urban	499.717	0.878	Rural to Urban	Rural origin
B.1.234	65708-Rural	65810-Urban	1089.060	0.940	Rural to Urban	Rural origin
B.1.234	65742-Urban	65761-Rural	205.562	0.748	Urban to Rural	Urban origin
B.1.234	Not MO	65536-Rural	32364.895	1.000	Not MO to Rural	Not MO origin
B.1.234	65747-Rural	65612-Urban	1089.060	0.940	Rural to Urban	Rural origin
B.1.234	Not MO	65616-Rural	10742.096	0.994	Not MO to Rural	Not MO origin
B.1.234	Not MO	65668-Rural	32364.895	1.000	Not MO to Rural	Not MO origin
B.1.234	65781-Urban	65673-Rural	1957.834	0.966	Urban to Rural	Urban origin
B.1.234	Not MO	65708-Rural	2247.425	0.970	Not MO to Rural	Not MO origin

B.1.234	Not MO	65734-Rural	554.431	0.889	Not MO to Rural	Not MO origin
B.1.234	Not MO	65781-Urban	407.670	0.855	Not MO to Urban	Not MO origin
B.1.240	Not MO	Other MO	365.905	0.921	Not MO to Other MO	Not MO origin
B.1.2 SubsetA	65610-Urban	65738-Urban	302.588	0.832	Urban to Urban	Urban origin
B.1.2 SubsetA	65616-Rural	65667-Rural	297.981	0.829	Rural to Rural	Rural origin
B.1.2 SubsetA	65706-Urban	65807-Urban	284.837	0.823	Urban to Urban	Urban origin
B.1.2 SubsetA	65740-Rural	65746-Urban	167.596	0.732	Rural to Urban	Rural origin
B.1.2 SubsetA	65742-Urban	65803-Urban	454.760	0.881	Urban to Urban	Urban origin
B.1.2 SubsetA	65742-Urban	65804-Urban	28322.199	1.000	Urban to Urban	Urban origin
B.1.2 SubsetA	65742-Urban	65616-Rural	7034.572	0.991	Urban to Rural	Urban origin
B.1.2 SubsetA	65742-Urban	65740-Rural	28322.199	1.000	Urban to Rural	Urban origin
B.1.2 SubsetA	65804-Urban	65802-Urban	209.016	0.773	Urban to Urban	Urban origin
B.1.2 SubsetB	65536-Rural	65742-Urban	216.589	0.830	Rural to Urban	Rural origin
B.1.2 SubsetB	65738-Urban	65753-Urban	118.621	0.728	Urban to Urban	Urban origin
B.1.2 SubsetB	Not MO	65738-Urban	169.154	0.792	Not MO to Urban	Not MO origin
B.1.2 SubsetB	Other MO	Not MO	442.482	0.909	Other MO to Not MO	Other MO origin
B.1.2 SubsetC	65605-Rural	65772-Urban	144.271	0.769	Rural to Urban	Rural origin
B.1.2 SubsetC	65605-Rural	65781-Urban	231.453	0.842	Rural to Urban	Rural origin
B.1.2 SubsetC	65738-Urban	Other MO	1138.976	0.963	Urban to Other MO	Urban origin
B.1.2 SubsetC	65738-Urban	65619-Urban	106.181	0.710	Urban to Urban	Urban origin
B.1.2 SubsetC	Not MO	65646-Urban	223.925	0.838	Not MO to Urban	Not MO origin
B.1.2 SubsetC	65807-Urban	65686-Rural	153.744	0.780	Urban to Rural	Urban origin
B.1.2 SubsetC	65809-Urban	65723-Rural	271.337	0.862	Urban to Rural	Urban origin
B.1.2 SubsetC	Not MO	65738-Urban	38971.884	1.000	Not MO to Urban	Not MO origin
B.1.2 SubsetC	65804-Urban	65769-Rural	514.058	0.922	Urban to Rural	Urban origin
B.1.2 SubsetC	65809-Urban	65803-Urban	38971.884	0.999	Urban to Urban	Urban origin
B.1.2 SubsetC	Not MO	65804-Urban	19464.291	0.998	Not MO to Urban	Not MO origin
B.1.2 SubsetC	Not MO	65807-Urban	172.251	0.799	Not MO to Urban	Not MO origin
B.1.2 SubsetC	Not MO	65809-Urban	38971.884	1.000	Not MO to Urban	Not MO origin
B.1.2 SubsetD	65653-Rural	65737-Rural	254.825	0.849	Rural to Rural	Rural origin
B.1.2 SubsetD	65653-Rural	65804-Urban	315.913	0.875	Rural to Urban	Rural origin
B.1.2 SubsetD	65653-Rural	Other MO	1587.393	0.972	Rural to Other MO	Rural origin
B.1.2 SubsetD	65679-Rural	65689-Rural	123.365	0.731	Rural to Rural	Rural origin
B.1.2 SubsetD	65681-Rural	65708-Rural	105.873	0.700	Rural to Rural	Rural origin
B.1.2 SubsetD	65686-Rural	64015-Urban	216.348	0.827	Rural to Urban	Rural origin

B.1.2 SubsetD	65653-Rural	65631-Urban	129.880	0.741	Rural to Urban	Rural origin
B.1.2 SubsetD	65804-Urban	65633-Urban	250.476	0.847	Urban to Urban	Urban origin
B.1.2 SubsetD	Not MO	65634-Rural	141.078	0.757	Not MO to Rural	Not MO origin
B.1.2 SubsetD	Not MO	65653-Rural	40772.068	1.000	Not MO to Rural	Not MO origin
B.1.2 SubsetD	65757-Urban	65661-Urban	123.365	0.731	Urban to Urban	Urban origin
B.1.2 SubsetD	65721-Urban	65669-Urban	187.940	0.806	Urban to Urban	Urban origin
B.1.2 SubsetD	65721-Urban	65705-Urban	329.169	0.879	Urban to Urban	Urban origin
B.1.2 SubsetD	Not MO	65721-Urban	1655.421	0.973	Not MO to Urban	Not MO origin
B.1.2 SubsetE	Not MO	65717-Urban	2170.636	0.987	Not MO to Urban	Not MO origin
B.1.2 SubsetF	65653-Rural	65742-Urban	216.874	0.885	Rural to Urban	Rural origin
B.1.2 SubsetF	Not MO	65653-Rural	145.156	0.837	Not MO to Rural	Not MO origin
B.1.2 SubsetF	65807-Urban	65725-Urban	91.974	0.765	Urban to Urban	Urban origin
B.1.311	Not MO	Other MO	261.126	0.931	Not MO to Other MO	Not MO origin
B.1.509	Not MO	Other MO	66.614	0.725	Not MO to Other MO	Not MO origin
B.1	65605-Rural	65706-Urban	138.560	0.754	Rural to Urban	Rural origin
B.1	65616-Rural	65714-Urban	1057.870	0.959	Rural to Urban	Rural origin
B.1	65617-Urban	65807-Urban	124.770	0.734	Urban to Urban	Urban origin
B.1	65625-Rural	65656-Rural	153.807	0.772	Rural to Rural	Rural origin
B.1	65625-Rural	65672-Rural	4489.961	0.990	Rural to Rural	Rural origin
B.1	Not MO	Other MO	13560.488	0.997	Not MO to Other MO	Not MO origin
B.1	65775-Rural	65020-Rural	623.835	0.932	Rural to Rural	Rural origin
B.1	Not MO	65201-Urban	161.892	0.781	Not MO to Urban	Not MO origin
B.1	65616-Rural	65605-Rural	506.284	0.918	Rural to Rural	Rural origin
B.1	Not MO	65616-Rural	40772.068	1.000	Not MO to Rural	Not MO origin
B.1	Other MO	65653-Rural	196.221	0.812	Other MO to Rural	Other MO origin
B.1	Other MO	65803-Urban	283.870	0.862	Other MO to Urban	Other MO origin

Transmission links filtered by Bayes factor ≥ 30 and posterior probability ≥ 0.7. Not MO, publicly available Missouri sequences from GISAID; Other MO, publicly available non-Missouri sequences from GISAID.

Supplementary Table 3. SARS-CoV-2 sites on ORF1a, ORF1b, N proteins undergoing pervasive positive selection

Gene	Position	ORF1ab Position	Posterior Probability	Bayes Factor
NSP3	175	ORF1a:993	0.987	107.976
NSP3	702	ORF1a:1520	0.987	103.935
NSP3	764	ORF1a:1582	0.983	82.627
NSP3	961	ORF1a:1779	0.984	88.594
NSP3	1105	ORF1a:1923	0.905	13.454
NSP3	1241	ORF1a:2059	0.979	66.794
NSP3	1788	ORF1a:2606	0.958	32.149
NSP4	458	ORF1a:3221	0.967	42.337
NSP8	145	ORF1a:4087	0.908	11.652
NSP12	161	ORF1b:161	0.956	28.532
NSP12	275	ORF1b:275	0.976	54.837
NSP12	440	ORF1b:440	0.906	12.788
NSP13	399	ORF1b:1322	0.963	33.502
NSP13	500	ORF1b:1423	0.956	27.756
NSP13	586	ORF1b:1509	0.91	12.960
ORF7a	47	NA	0.913	12.017
ORF7a	84		0.905	10.920
N	67	NA	1.000	3.060E+13
N	194		1.000	3.612E+05
N	199		1.000	1.074E+06
N	205		1.000	2.488E+03
N	207		0.962	3.020E+01
N	371		1.000	2.945E+12
N	377		1.000	6.699E+07
N	391		1.000	1.175E+13

Positively selected sites determined as posterior probability > 0.9 using Fast, Unconstrained Bayesian AppRoximation for Inferring Selection Assumption (FUBAR) method. *, amino acids associated with Missouri-origin lineages.

Supplementary Table 4. Positive selection analysis at position N:204 and N gene-wide analysis. Site-specific positive selection was determined as posterior probability > 0.9 using the Fast, Unconstrained Bayesian AppRoximation for Inferring Selection Assumption (FUBAR) method in HyPhy. -, No evidence for site-specific positive selection. Gene-wide positive selection was determined as p-value < 0.05 using the Branch-site Unrestricted Statistical Test for Episodic Diversification (BUSTED) method.

	Rural						Urban						Total					
**Month**	07-2020	08-2020	09-2020	10-2020	11-2020	12-2020	07-2020	08-2020	09-2020	10-2020	11-2020	12-2020	07-2020	08-2020	09-2020	10-2020	11-2020	12-2020
**Evidence for site-specific pervasive diversifying selection at N:204**																		
Alpha	-	-	-	3.158	-	2.964	3.634	-	-	-	-	3.813	3.099	-	-	2.886	-	2.501
Beta	-	-	-	27.202	-	25.833	30.683	-	-	-	-	32.926	27.389	-	-	31.157	-	22.356
Posterior Probability	-	-	-	0.933	-	0.933	0.934	-	-	-	-	0.936	0.935	-	-	0.955	-	0.931
Bayes Factor	-	-	-	16.075	-	16.460	16.573	-	-	-	-	17.280	17.036	-	-	23.579	-	16.489
**Evidence for gene-wide episodic or pervasive diversifying selection**																		
p-value	0.0168	0.353	0.477	0.295	0.406	0.500	0.500	0.144	0.399	0.087	0.285	0.482	0.500	0.315	0.113	0.00470	0.274	0.500
dN/dS	309.192	1.617	1.674	1.349	1.341	1.000	2.293	2.303	1.451	1.779	1.484	1.133	1.002	1.786	2.149	2.045	1.467	2.636
Evidence of episodic diversifying selection	Yes	No	No	No	No	No	No	No	No	No	No	No	No	No	No	Yes	No	No

Supplementary Table 5. Frequency of substitutions N:R203K/G204R among each lineage among study samples. Highlighted residues indicate an amino acid substitution. Lineages were assigned by the Phylogenetic Assignment of Named Global Outbreak Lineages (PANGOLIN) software (PANGO v4.0.6 (2022-04-22)).

Pango Lineage	Residue 203	Residue 204	Frequency	Proportion of lineage sequences containing N:R203K/G204R (%)
B.1.1.337	**K**	**R**	75	96%
B.1.1.337	R	G	3	0%
B.1.1	**K**	**R**	50	96%
B.1.1	R	G	2	0%
B.1.1.33	**K**	**R**	5	100%
B.1.1.135	**K**	**R**	4	100%
B.1.1.432	**K**	**R**	4	100%
C.23	**K**	**R**	4	100%
B.1.1.464	**K**	**R**	2	100%
B.1.1.225	**K**	**R**	1	100%
B.1.1.291	**K**	**R**	1	100%
B.1.1.304	**K**	**R**	1	100%
B.1.1.376	**K**	**R**	1	100%
B.1	R	G	66	0%
B.1.110.3	R	G	3	0%
B.1.139	R	G	7	0%
B.1.162	R	G	1	0%
B.1.199	R	G	1	0%
B.1.2	R	G	406	0%
B.1.206	R	G	1	0%
B.1.234	R	G	137	0%
B.1.240	R	G	45	0%
B.1.243	R	G	4	0%
B.1.265	R	G	3	0%
B.1.311	R	G	25	0%
B.1.324	R	G	11	0%
B.1.340	R	G	2	0%
B.1.349	R	G	1	0%
B.1.361	R	G	1	0%
B.1.369	R	G	6	0%
B.1.377	R	**R**	8	0%
B.1.390	R	G	2	0%
B.1.396	R	G	1	0%
B.1.400	R	G	6	0%
B.1.413	R	G	5	0%
B.1.501	R	G	1	0%
B.1.509	R	G	29	0%
B.1.541	R	G	1	0%
B.1.544	R	G	5	0%

B.1.556	R	G	1	0%
B.1.564	R	G	2	0%
B.1.565	R	G	5	0%
B.1.568	R	G	2	0%
B.1.569	R	G	2	0%
B.1.574	R	G	1	0%
B.1.576	R	G	1	0%
B.1.577	R	G	8	0%
B.1.578	R	G	1	0%
B.1.582	R	G	3	0%
B.1.591	R	G	4	0%
B.1.595	R	G	11	0%
B.1.595.2	R	G	1	0%
B.1.596	R	G	3	0%
B.1.609	R	G	2	0%
B.4	R	G	1	0%

Supplementary Figures

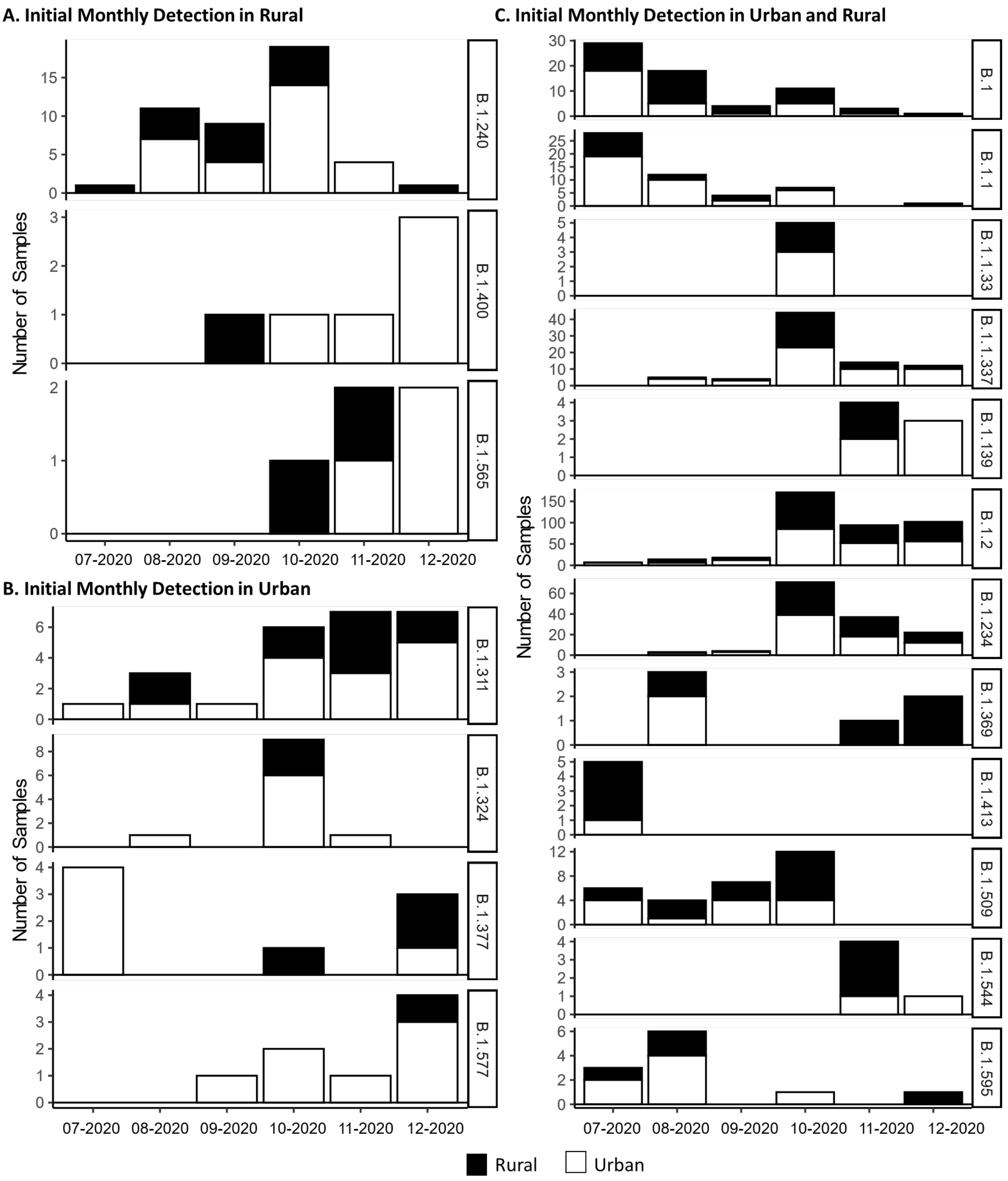

Supplementary Figure 1. Frequency of urban and rural cases by lineage. All Pango lineages with at least three study samples are shown with monthly urban and rural comparison. A) Pango lineages first detected among rural samples. B) Pango lineages first detected among urban samples. C) Pango lineages first detected in both urban and rural samples.

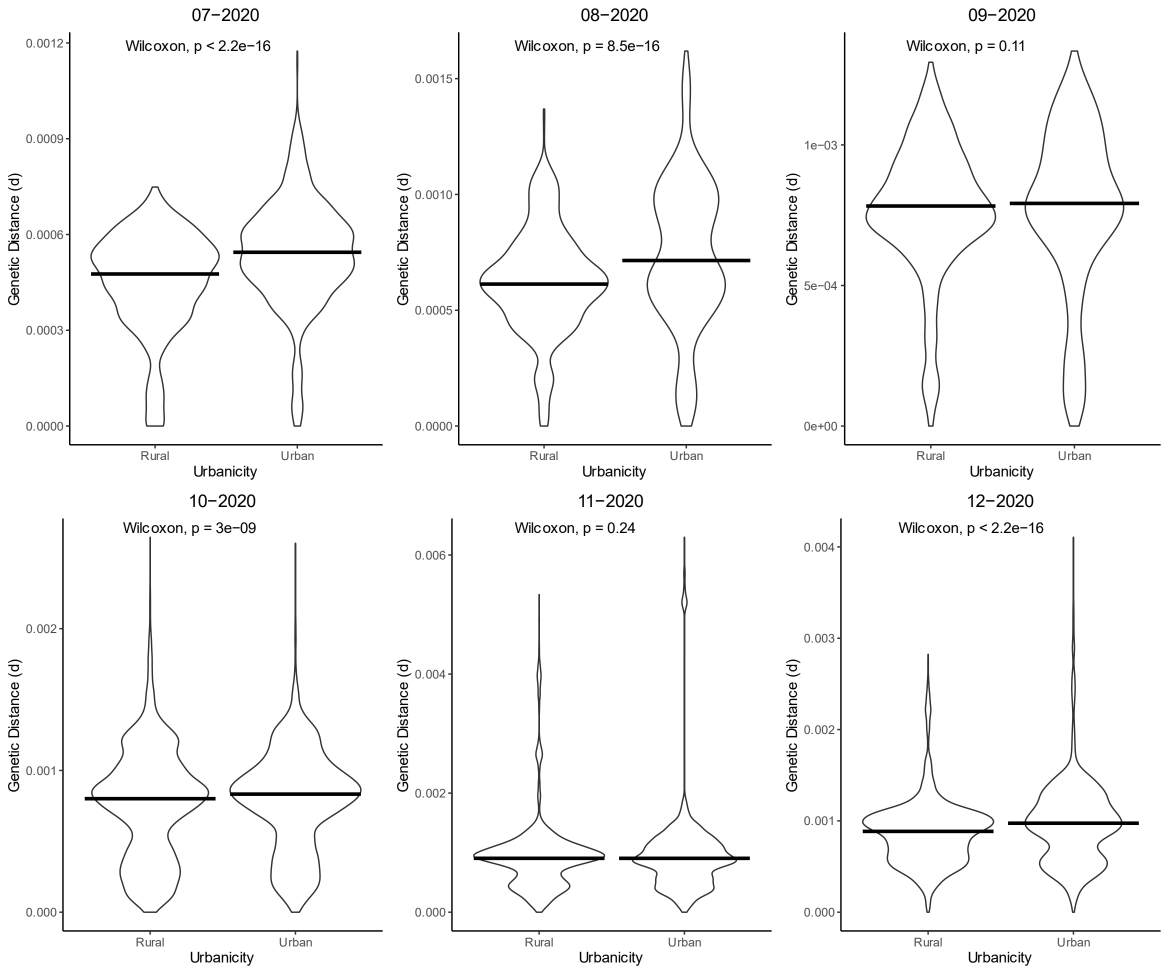

Supplementary Figure 2. Monthly Viral Genetic Diversity Across Urbanicity. Each violin plot represents the distribution of pairwise genetic distances between all urban and all rural sequences collected from each month. The bold horizontal lines represent the median pairwise genetic distance (d) among all samples in each population. A significant difference in genetic diversity between urban and rural sequences was determined with a p-value < 0.05.

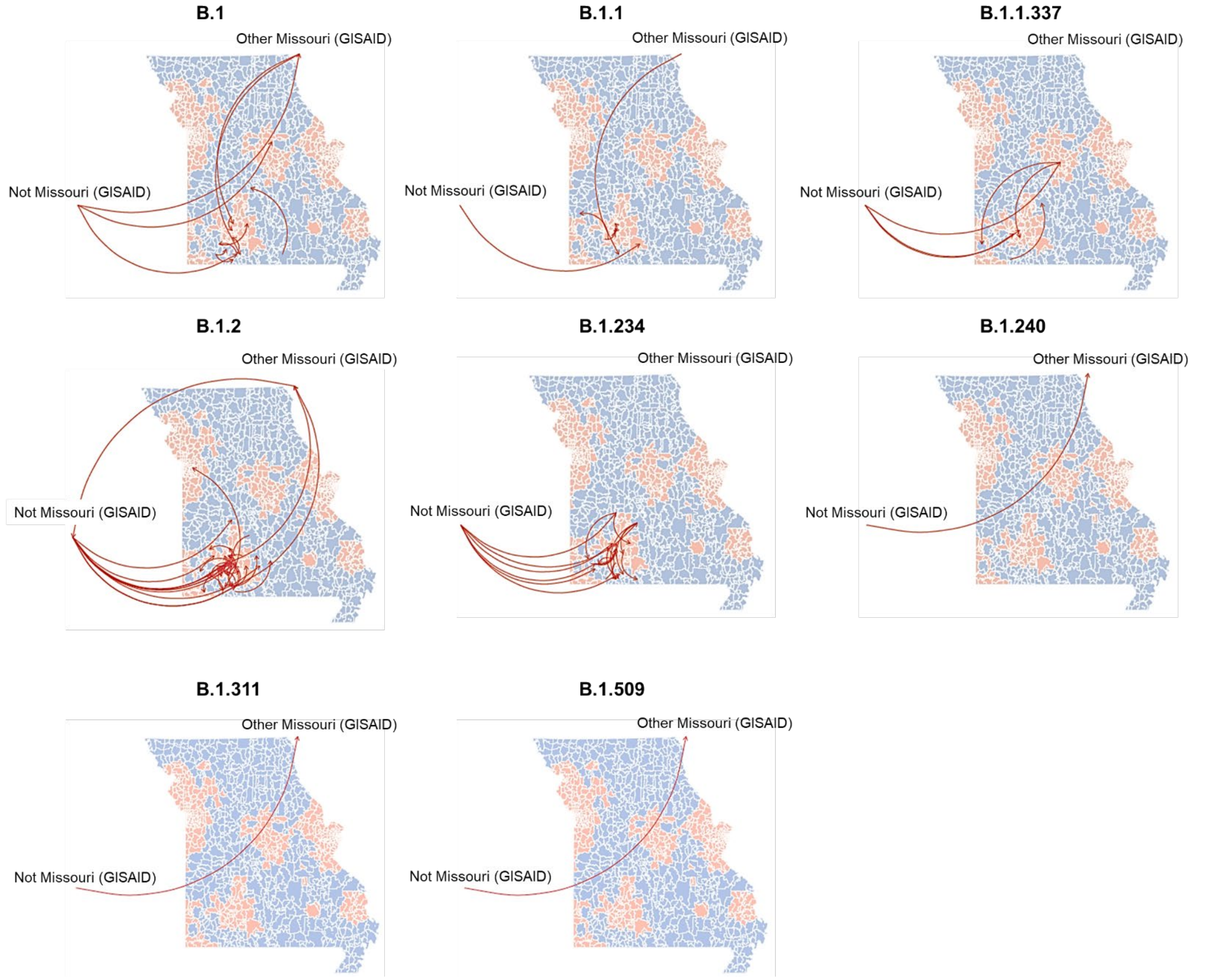

Supplementary Figure 3. Transmission links across Southwest Missouri. Maps of transmission links for each lineage containing at least 10 urban and 10 rural samples. Red lines represent all possible transmission links defined as Bayes factor ≥ 3 and posterior probability ≥ 0.7. Arrowheads represent direction of transmission link. A Bayesian Stochastic Search Variable Selection (BSSVS) was used for phylogeographic analyses.

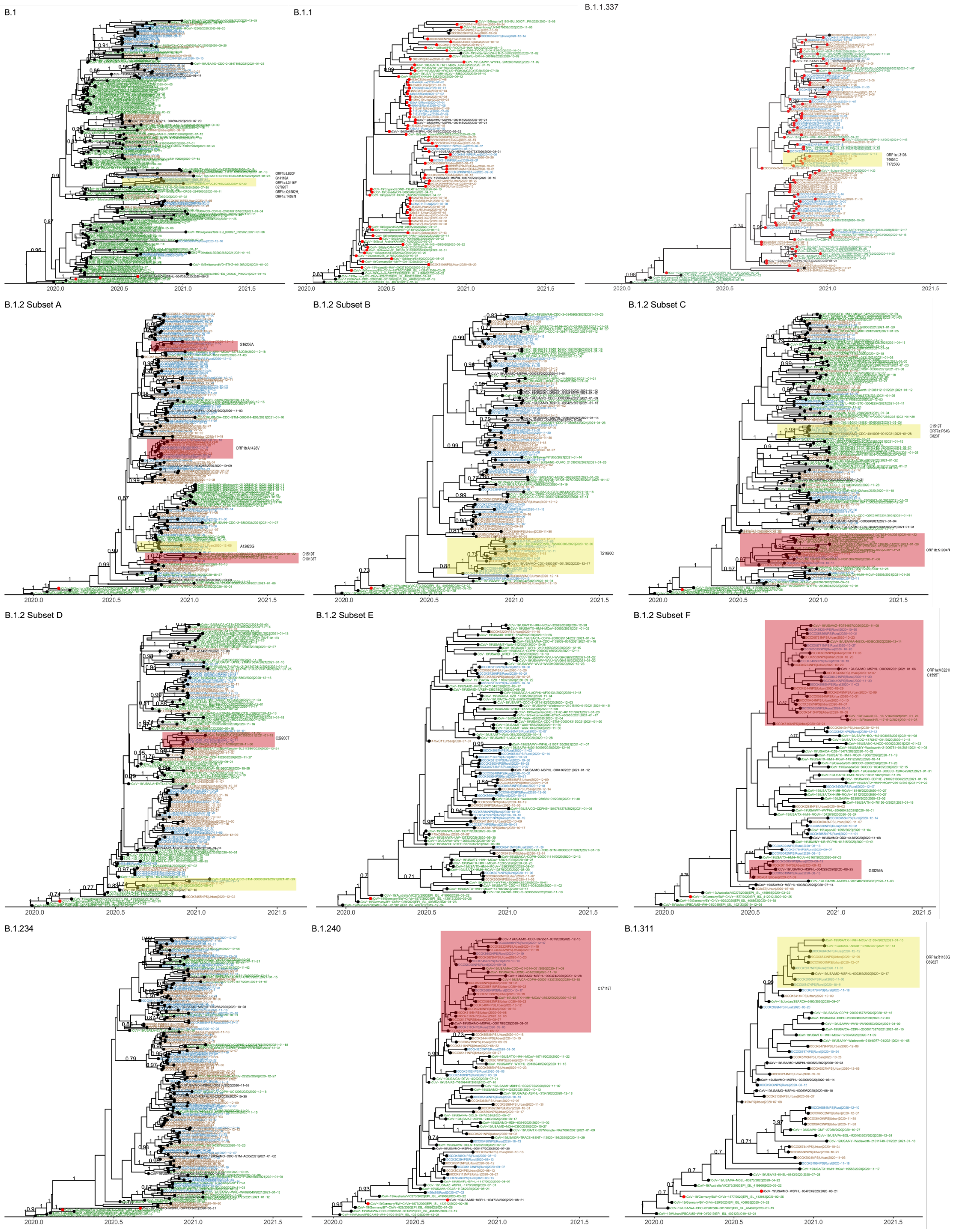
B.1
B.1.1
B.1.1.337
B.1.2 Subset A
B.1.2 Subset B
B.1.2 Subset C
B.1.2 Subset D
B.1.2 Subset E
B.1.2 Subset F
B.1.234
B.1.240
B.1.311
2020.0
2020.5
2021.0
2021.5

B.1.509

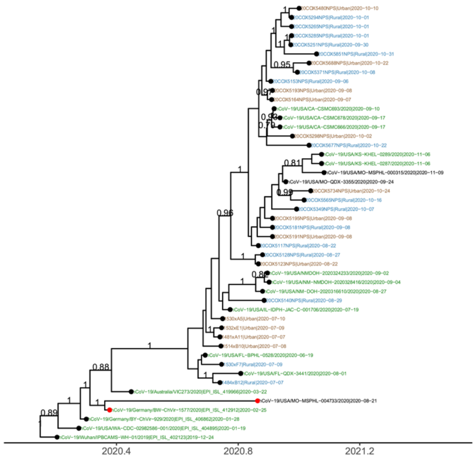

Supplementary Figure 4. Time-scaled phylogenetic trees of each major lineage identified in Southwest Missouri. Branches with posterior probabilities of ≥ 0.70 are labeled. Public sequences, represented as black (GISAID Missouri) and green (GISAID not Missouri) tip labels, are sequences downloaded from GISAID and identified as the sequences with the smallest distance from our study sequences using the complete composition vector method. Study sequences are represented in blue (rural) and brown (urban). Tip points indicate N:R203K/G204R: red, contains both substitutions; black, does not have these substitutions. Rural lineages are annotated in transparent yellow boxes as defined lineages containing at least 5 samples with an earliest detected sample from a rural study sample and a posterior probability of ≥ 0.7. Likewise, urban lineages are annotated in transparent red boxes.

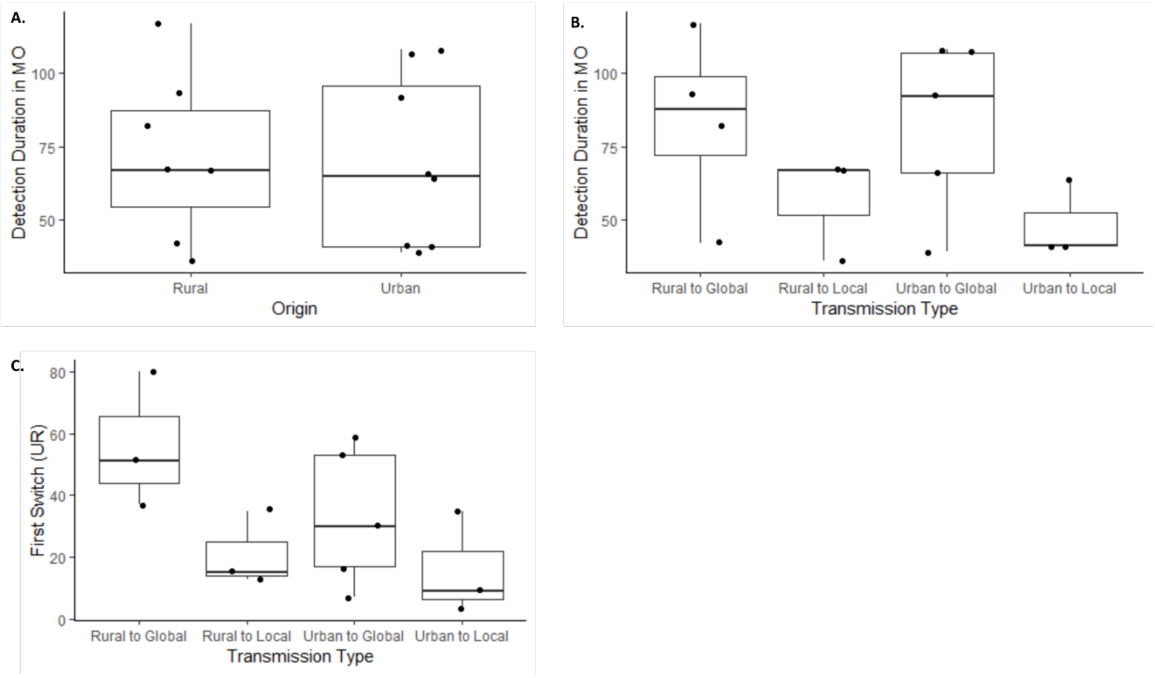

Supplementary Figure 5. Duration of detection for each lineage that emerged from Missouri. Overall, Missouri-origin lineages were detected among our study samples for an average of 2 months. A) Comparison of duration of detection between all rural and all urban lineages. B) Comparison of duration of detection from date of first detection to date of first detection in a global (non-Missouri) or local (other Missouri) sample. Durations of detection were calculated based on the first and last collection dates associated with each lineage. C) Comparison of duration from the date of first detection to the next date associated with a global sample or the next date associated with a sample associated with the alternative urbanicity. Time to “First Switch” for a “Rural to Local” transmission type would be calculated using the first date of detection in the rural sample and the date of the first urban sample within that lineage. Likewise, for an “Urban to Local” transmission type, time to “First Switch” would be calculated using the first date of detection in the urban sample and the date of the first rural sample within that lineage. Each dot represents an individual lineage and were jittered for visualization, boxes represent the interquartile ranges, the line within each box represented the median, and whiskers represent minimum and maximum values. All durations are reported in days. MO, Missouri.
